# Differences in Workloads of Maximal Tasks in Active-Duty Firefighters

**DOI:** 10.3390/healthcare12151495

**Published:** 2024-07-27

**Authors:** Rudi A. Marciniak, Carly A. Wahl, Kyle T. Ebersole

**Affiliations:** 1 School of Kinesiology, Ball State University, Muncie, IN 47306, USA; 2 Department of Kinesiology, Sport, and Recreation, Eastern Illinois University, Charleston, IL 61920, USA; cawahl@eiu.edu; 3 School of Rehabilitation Sciences & Technology, University of Wisconsin-Milwaukee, Milwaukee, WI 53211, USA; ebersole@uwm.edu

**Keywords:** occupational athlete, firefighter, workload, job demands, exercise testing, fitness, health, readiness

## Abstract

The purpose of this study was to compare the workload of a maximal treadmill test (TREAD) and a fire suppression task (BURN) in firefighters and to examine their relationships to fitness as measured by body mass index (BMI), percent body fat (BF%), and peak aerobic capacity (VO_2PEAK_). The amount of time spent in the heart rate (HR) intensity ranges of 50–59% HR_MAX_ (ZONE1), 60–69% HR_MAX_ (ZONE2), 70–79% HR_MAX_ (ZONE3), 80–89% HR_MAX_ (ZONE4), and ≥90% HR_MAX_ (ZONE5) quantified the workload as the Edward’s Training Impulse for TREAD (ETRIMP_TREAD_) and BURN (ETRIMP_BURN_). The ETRIMP_TREAD_ was significantly less than ETRIMP_BURN_. For TREAD, ZONE5 > ZONE2 and ZONE3. For BURN, ZONE4 > ZONE1, ZONE2, and ZONE5 > ZONE1, ZONE2, and ZONE3. A lower BF% and greater VO_2PEAK_ were related to a greater ETRIMP_TREAD_ and unrelated to ETRIMP_BURN_. For BURN only, a lower BF% and greater VO_2PEAK_ were related to less time in ZONE5. BMI was unrelated to all workload measures. Laboratory-based maximal exercise testing does not adequately reflect the workload of simulated fire suppression and therefore may not be indicative of firefighter readiness to meet job demands. Less-fit firefighters rely on higher cardiovascular intensities to complete the same workload, and practitioners should consider this when selecting strategies to reduce job-associated cardiovascular risk.

## 1. Introduction

The job demands of firefighting are highly unpredictable and physically strenuous, where the risk of injury is nearly two times greater than other occupations in the United States [1]. Firefighters (FFs) respond to various types of emergencies, including medical and fire emergencies, and are disproportionally at risk for injuries on the fireground [2], despite only 5% of job demands including fire suppression [3]. In 2022, 33% of all reported FF injuries occurred on the fireground, and the leading cause of such injuries was overexertion or strain [4]. Moreover, overexertion and strain accounted for over half (54%) of on-duty deaths in 2023 [5]. The consequences of these injuries are multifactorial, ranging from personal impacts to the affected FF(s) to economic impacts, with the national cost estimated to range between USD 1.6 and USD 5.9 billion annually [6]. To mitigate such exertional injuries and to reduce the burden of the resultant healthcare and FF recovery, enhancing the quantification of job demands and identifying health and fitness characteristics that increase workload is paramount to the creation of appropriate fitness programs.

Measures of health and fitness, such as greater aerobic capacity [7] and lower levels of obesity [2,8], have been generally linked to readiness for duty and reduced injury risk among FFs. The National Fire Protection Association (NFPA) suggests FFs meet a minimum aerobic capacity (VO_2PEAK_) of 42 mL/kg/min to adequately complete the strenuous demands of firefighting [9]. As it relates to job readiness, a greater VO_2PEAK_ has been linked to quicker completion times during simulated fireground testing [10] and candidate physical ability tests [11]. FFs with VO_2PEAK_ capacities below 43 mL/kg/min are 2.2 times more likely to sustain injuries, when compared to individuals with capacities that exceed 48 mL/kg/min [12]. Furthermore, obese FFs are approximately three times more likely to experience an on-duty cardiac fatality [13]. This statistic is alarming, as up to 75% of FFs may be considered overweight or obese when measured by body mass index (BMI) or percent body fat (BF%) [14,15] and average FF weight is predicted to climb with increasing age [16]. The aforementioned information is additionally concerning, as the risk of not meeting the NFPA VO_2PEAK_ recommendation is 3.3 times greater for overweight FFs [17]. As such, FFs with a lower VO_2PEAK_, especially those with greater obesity, are less likely to be ready for duty, in addition to being at a heightened risk for incurring an injury.

To maximize the physical capabilities necessary to meet FF job demands and reduce risk of injury, the Fire Service Joint Labor Management Wellness–Fitness Initiative (WFI) was established to support the routine measurement and maintenance of FF health through measures like aerobic capacity and body composition [18]. In a non-punitive manner, fire departments estimate FF VO_2PEAK_ through submaximal WFI graded treadmill tests and body composition through skinfold measurements [18]. The WFI graded treadmill test has also been demonstrated to accurately estimate FF VO_2PEAK_ when utilized as a maximal aerobic capacity test [19], and there is potential for its use as a surrogate test of readiness to meet maximal on-duty demands like that of fire suppression calls [20]. However, while aerobic capacity-testing has been historically utilized to reflect FF health and wellness, it is possible that the physiological responses to laboratory-based testing may not indicate the expected physiological responses to field-based tasks such as fire suppression. It is important to identify if physiological differences exist between the maximal laboratory- and field-based tasks, as it is likely that the health and fitness of most active-duty FFs are examined annually after hire [9]. Though most FFs are likely to complete routine fire suppression training exercises to regularly engage the skills necessary to be performed when a fire emergency call occurs (i.e., use of personal protective equipment, heat exposure, etc.), the physiological response to such field-work is often not included in annual FF examinations [9]. If the workload expectations of a maximal treadmill test commonly utilized in the fire service and the maximal physiological responses experienced in a field-based task are reflective of each other, an annual treadmill test may provide insight into FF readiness for duty beyond simply indicating FF health and fitness levels.

Recently, researchers have attempted to quantify the field-based demands required of FFs through the use of training load measures that are an established practice within strength and conditioning science among sport athlete populations [21] and are linked to overexertion-based injuries [22,23] similar to those experienced by FFs. The internal load of a task is quantified by the intrinsic responses initiated by completing objective work [21,24,25]. An established internal load measure that utilizes the accumulated time in five arbitrary heart rate zones and sums the product of the time spent in each zone throughout a task by weighted factors is termed Edward’s Training Impulse (ETRIMP) [26]. ETRIMP is demonstrated to accurately quantify the internal load of exercise across varying intensities and mixed modalities [27]; therefore, it is likely to be well suited for quantifying the load of field-based firefighting tasks. Training load measures have been utilized to monitor exertional exposures and their affiliated responses, including changes in stress [28] and fatigue status [29], as well as to predict the risk of over-use injuries [30,31,32] and illness [33] in athletes. Accordingly, the use of such measures can enhance the identification of injury risk and decision making to avoid possible injury [21], and are also suggested to partially explain performance outcomes in response to training [21,34,35]. Exploring the use of such metrics in occupational settings, especially within the fire service, where over-exertion is prevalent, may inform best practices for fitness and/or injury prevention programming, to maximize occupational performance while minimizing the affiliated health risks. 

The application of load measures has recently been applied to FF populations to attempt a quantification of occupational *workload* demands. To date, the on-duty FF workload using a heart rate-based measure has been quantified for several on-duty job tasks [25], and recently, the ETRIMP of on-duty responses to fire emergencies that included fire suppression and/or auto-extrication were demonstrated to be six times more physiologically demanding than other on-duty fire and medical call responses [20]. However, the workload elicited by fire suppression in a training setting has not been examined, and it remains unclear if the workload of a training fire is accurately represented by a maximal laboratory-based test. Further, it is unclear how health and fitness measures like aerobic capacity and body composition may influence the resulting workload in a laboratory-based test or in a live-fire training scenario. Since laboratory tests are often used to inform job readiness and simulated field-based tasks are utilized as training modalities, it is important to determine if these tasks reflect similar workloads and/or if individual health and fitness characteristics uniquely influence the demands of each task. Therefore, the purpose of this study was to compare the workload (i.e., ETRIMP) of a maximal laboratory-based treadmill test and a field-based fire suppression task, including an examination of the distribution of time spent in intensity zones, as well as to examine the relationships between the elicited loads and measures of health and fitness. Due to the previous literature establishing that heart rate responses during fire suppression are greater than during laboratory-based testing [36], it was hypothesized that the ETRIMP of a fire suppression task would be greater than a maximal treadmill test. Additionally, it was hypothesized that the workloads of both tasks would be related to the VO_2PEAK_ and BF%, but unrelated to BMI, due to the prior literature suggesting that BMI is unrelated to cardiovascular responses during [37], and when recovering from [38], exercise in FFs.

## 2. Materials and Methods

### 2.1. Participants

Fifteen active-duty career FFs (13 males, 2 females) from a fire department in a metropolitan, midwestern city in the United States volunteered to participate in this study (mean ± SD; age = 34.40 ± 7.38 yrs; yrs of exp = 9.27 ± 6.67 yrs; height = 179.49 ± 6.20 cm; body mass = 91.76 ± 17.24 kg). The criteria for inclusion consisted of participants needing to be cleared for full, active duty by their department. Participants were informed that they could withdraw from the study at any time without consequences from the research team or their respective fire department. The Institutional Review Board at the University of Wisconsin–Milwaukee approved all components of this research study. All participants gave written informed consent before data collection.

### 2.2. Procedures

The first study session was completed in a laboratory-based setting in a firehouse of the participating department while the participants were off-duty. At this session, body mass was measured and recorded to the nearest 0.1 kg, and height was measured and recorded to the nearest 0.5 cm. From these measures, the BMI was calculated for each participant (kg/m^2^). In accordance with procedures described by the American College of Sports Medicine (ACSM) [39], body density was then calculated using the Jackson and Pollock three-site skinfold method. For males, measures were taken at the triceps, subscapular, and pectoral locations, and for females, measures were taken at the triceps, abdominal, and suprailiac locations. All skinfold sites were measured to the nearest millimeter (mm) by the same investigator using a Lange skinfold caliper (Beta Technology, Santa Cruz, CA, USA). From the quantified body densities, the BF% was calculated using the Siri body fat equation [39]. Participants were then fitted with a sealed mask that carried their expired air to a portable metabolic analysis unit (COSMED, Rome, Italy) that collected their breath-by-breath oxygen consumption while completing a maximal graded treadmill exercise test (TREAD) [19] in exercise clothes. The TREAD protocol began with a 3-minute walking warm-up at 3.0 mi/h (4.83 km/h) and 0% grade, followed by an increase to 4.5 mi/h (7.24 km/h) for 1 min. The protocol then involved alternating increases of a 2% grade and 0.5 mi/h (0.80 km/h) every minute until maximal effort was exerted. The TREAD test was terminated when at least two of the following three criteria were achieved: (a) meeting or exceeding a target maximal heart rate (HR) for more than 15 s, (b) the participant’s rating of perceived exertion was ≥17 from Borg’s 6–20 scale, and/or (c) volitional termination by the participant due to fatigue. The target maximal HR (HR_MAX_) was defined as 100% of the age-predicted maximum HR from the following equation: HR_MAX_ = 208 − (0.7 × age) [40]. Participant VO_2PEAK_ was quantified as the peak rate of oxygen consumption (mL/kg/min) achieved during the treadmill protocol at the time of test termination. TREAD duration was quantified as the time from when the protocol began until the time the test was terminated. 

The second study session was completed in the burn tower of the participating department’s training academy while on-duty, yet prior to responding to any emergency calls for the shift. The apparatus of each participating crew was taken “out of service” for the duration of the session to avoid any emergency call response interruptions. At this session, participants completed a fire suppression activity (BURN) while donning a full personal protective ensemble (PPE) and breathing from a self-contained breathing apparatus (SCBA). Participants were instructed to perform the task at a pace as comparable as possible to the expectations of a live fire suppression (i.e., maximal effort) and knew in advance the fire was located in the attic (i.e., third floor) of the burn tower. A non-participant FF used a department-issued handheld thermal imager to measure the temperature inside the attic environment at shoulder height for all evolutions (average: Start [173.22 °C], End [112.94 °C]). In randomized groups of three, participants breached a ground-level door to a burn tower, advanced the hose to a fire on the third floor of the tower, suppressed the fire, completed a victim search of the area, and retreated from the tower. The specific tasks that were completed by participants in the BURN were not recorded across the timeline to allow for identification of what each did and when. However, the skills used to accomplish the task were completed as they would, and in the order they would be in the field. All firefighting tasks were completed within the parameters that were expected in the field. The duration of the BURN was quantified as the time the door was breached until the time all three team members exited the burn tower.

#### 2.2.1. Edward’s Training Impulse

The ETRIMP was calculated post hoc to quantify the physiological workload across TREAD and BURN. Specifically, the total time (min) spent in each HR zone for each task duration was multiplied by a weighting factor and summed to quantify a total ETRIMP score [26]. The five HR zones were ranges of percentages of the estimated HR_MAX_ (%HR_MAX_), including 50–59% HR_MAX_, 60–69% HR_MAX_, 70–79% HR_MAX_, 80–89% HR_MAX_, and ≥90% HR_MAX_. For TREAD and BURN, the HR data per second was marked post hoc into one of the five HR zones and then summed into the duration (HH:MM:SS) spent in each zone for each task. The time spent in each HR zone was then multiplied by the zone’s weighting factor (i.e., 50–59% HR_MAX_ = 1, 60–69% HR_MAX_ = 2, 70–79% HR_MAX_ = 3, 80–89% HR_MAX_ = 4, and ≥90% HR_MAX_ = 5) and summed to quantify the ETRIMP for TREAD (ETRIMP_TREAD_) and BURN (ETRIMP_BURN_).

#### 2.2.2. Intensity Zones

To quantify the percent of total time spent in each HR zone utilized to calculate ETRIMP_TREAD_ and ETRIMP_BURN_, the summed, non-weighted time in each zone was divided by the total task duration, resulting in a percentage of time at 50–59% HR_MAX_ (ZONE1), 60–69% HR_MAX_ (ZONE2), 70–79% HR_MAX_ (ZONE3), 80–89% HR_MAX_ (ZONE4), and ≥90% HR_MAX_ (ZONE5).

### 2.3. Statistical Analyses

Prior to statistical analysis, the normality of the data was confirmed with visual observation of univariate histograms and Q-Q plots. Assumptions of sphericity were also confirmed using Mauchly’s test of sphericity. A paired *t*-test examined for differences between ETRIMP_TREAD_ and ETRIMP_BURN_. A 2 × 5 (TEST × ZONE) within-subjects repeated measures analysis of variance (RM ANOVA) was performed to examine for differences in the zones for both tests. A follow-up analysis included least significant differences for the comparison of simple effects with Bonferroni adjustments. Relationships between ETRIMP_TREAD_, ETRIMP_BURN_, and all TREAD and BURN zones with the health and fitness measures (i.e., BMI, BF%, and VO_2PEAK_) were examined utilizing bivariate Pearson correlations.

All statistical analyses were conducted using IBM SPSS 28 statistical software (IBM Corp., Armonk, NY, USA), and an alpha of 0.05 was used to determine statistical significance for all analyses. Additionally, standardized effect-size statistics were calculated to determine potential practical differences between the TEST for the ETRIMP and zones. Given the small sample size, Hedge’s *g* effect-size statistics were chosen [41,42] and interpreted using the following criteria [43]: *trivial* (*g* ≤ 0.19), *small* (0.20 ≤ *g* ≤ 0.49), *medium* (0.50 ≤ *g* ≤ 0.79), *large* (0.80 ≤ *g* ≤ 1.29), and *very large* (*g* ≥ 1.30).

## 3. Results

**Figure 1:**
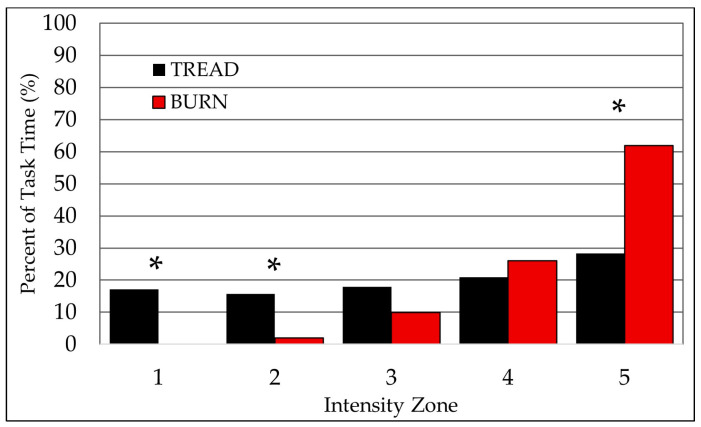
Distribution of time across workload intensity zones for laboratory- and field-based tasks. *, significant difference (*p* < 0.05) between tasks.

The group average (mean ± SD) HR responses throughout the TREAD and BURN tasks were 77.59 ± 0.04% and 91.05 ± 0.06%, respectively. Subsequently, the paired *t*-test demonstrated a very large significant difference in overall ETRIMP, where ETRIMP_TREAD_ < ETRIMP_BURN_ (*t* = −4.885, *p* < 0.001, *g* = 1.594; Table 1), despite TREAD and BURN requiring similar durations for completion (11.64 ± 1.90 = 11.01 ± 2.04 min, respectively; *t* = 1.049, *p* = 0.312). Skewness and kurtosis calculations and visual inspections of the normal Q-Q plots for the data revealed no consistent outliers across the dependent variables, which satisfied the normality assumption for the RM ANOVA and post hoc calculations. However, the assumption of sphericity was violated (Mauchly’s *W* = 0.027, *p* < 0.001), and a Greenhouse–Geisser correction was applied to the RM ANOVA. The 2 × 5 (TEST × ZONE) RM ANOVA demonstrated a significant interaction (*F*_4,1.711_ = 12.189, *p* < 0.001). Follow-up simple effect analyses for TEST (Figure 1) indicated very large significant differences in the time spent in several zones, where more time was spent in ZONE1 (*F*_1,14_ = 36.669, *p* < 0.001, *g* = 2.151) and ZONE2 (*F*_1,14_ = 26.524, *p* < 0.001, *g* = 1.790) for TREAD than BURN; however, less time was spent in ZONE5 (*F*_1,14_ = 15.626, *p* < 0.001, *g* = 1.340) for TREAD than BURN. There were non-significant mean differences between TREAD and BURN for ZONE3 (*F*_1,14_ = 3.421, *p* = 0.086, *g* = 0.613) and ZONE4 (*F*_1,14_ = 0.788, *p* = 0.390, *g* = 0.323). 

Follow-up pairwise analyses for ZONE (Table 1) indicated significant differences across zones for TREAD (*F*_4,11_ = 9.734, *p* = 0.001). Specifically, the amount of time spent in ZONE1 was not different from ZONE2 (*p* = 1.000, *g* = 0.146), ZONE3 (*p* = 1.000, *g* = 0.082), ZONE4 (*p* = 1.000, *g* = 0.410), or ZONE5 (*p* = 0.271, *g* = 1.092) for TREAD. Similarly, the amount of time spent in ZONE2 for TREAD was not different from ZONE3 (*p* = 1.000, *g* = 0.322) and ZONE4 (*p* = 1.000, *g* = 0.684), yet was significantly less than ZONE5 (*p* = 0.003, *g* = 1.421). TREAD did not elicit different amounts of time spent in ZONE3 and ZONE4 (*p* = 0.616, *g* = 0.628), but significantly less time was spent in ZONE3 than ZONE5 (*p* = 0.012, *g* = 1.563). Finally, TREAD did not elicit different amounts of time spent between ZONE4 and ZONE5 (*p* = 0.541, *g* = 0.970). 

The follow-up pairwise analyses for ZONE (Table 1) indicated significant differences across zones for BURN (*F*_3,12_ = 7.912, *p* = 0.004). Specifically, the amount of time spent in ZONE1 for BURN was not different from ZONE2 (*p* = 1.000, *g* = 0.447) or ZONE3 (*p* = 0.436, *g* = 0.788), yet was significantly less than ZONE4 (*p* = 0.003, *g* = 1.688) and ZONE5 (*p* < 0.001, *g* = 2.547). For BURN, ZONE2 was not different from ZONE3 (*p* = 0.339, *g* = 0.618), yet was significantly less than ZONE4 (*p* = 0.012, *g* = 1.540) and ZONE5 (*p* < 0.001, *g* = 2.453). The time spent in ZONE3 was not different from ZONE4 (*p* = 0.202, *g* = 0.806), yet was significantly less than ZONE5 (*p* = 0.011, *g* = 1.895). Finally, the amount of time spent in ZONE4 was not different from ZONE5 (*p* = 0.171, *g* = 1.244) for BURN.

**Figure 2:**
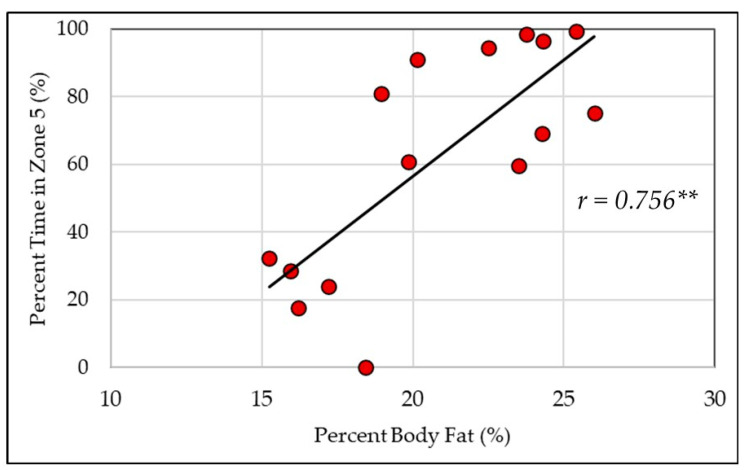
Percent body fat is positively related to time spent in Zone5 (≥90% HR_MAX_) during fire suppression activity. **, significant relationship (*p* < 0.001).

**Figure 3:**
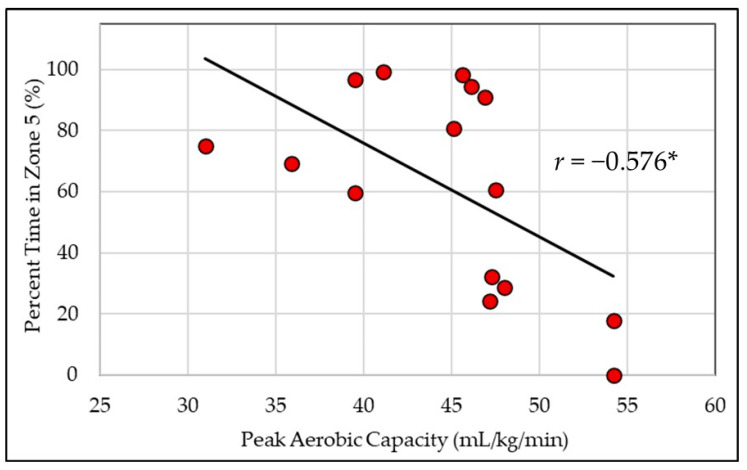
Peak aerobic capacity is negatively related to time spent in Zone5 (≥90% HR_MAX_) during fire suppression activity. *, significant relationship (*p* < 0.05).

All the correlation coefficients are presented in Table 2. ETRIMP_TREAD_ was significantly negatively related to BF% (20.78 ± 3.69%) and positively related to VO_2PEAK_ (44.61 ± 6.28 mL/kg/min)_,_ but non-significantly related to BMI (28.46 ± 5.14 kg/m^2^). In contrast, ETRIMP_BURN_ was non-significantly related to all fitness measures. For TREAD, BF% and VO2_PEAK_ were non-significantly related to all zones. For BURN, no time was spent in ZONE1 and therefore, correlations were not calculated for that zone. BF% was significantly negatively related to ZONE3_BURN_ and ZONE4_BURN_ and positively related to ZONE5_BURN_ (Figure 2). VO_2PEAK_ was significantly positively related to ZONE3_BURN_ and negatively related to ZONE5_BURN_ (Figure 3), but non-significantly related to ZONE2_BURN_ and ZONE4_BURN_. BMI was unrelated to all zones for TREAD and for BURN.

## 4. Discussion

A limited number of studies have quantified the workload of firefighting job tasks using an HR-based technique. Further, a direct comparison of the workload required of a maximal laboratory-based test and field-based task, and the health and fitness factors that may influence such workloads, remains unexamined. The results of this study demonstrated the workload of a field-based controlled fire suppression task to be greater than a laboratory-based maximal treadmill test, which is due to the suppression task eliciting significantly greater amounts of time at intensities near maximal limits compared to the laboratory-based test. Additionally, less-fit FFs spend significantly greater amounts of time at higher cardiovascular intensities than more-fit individuals during controlled fire suppression.

The comparison of task-specific workloads in this study demonstrated the ETRIMP_TREAD_ to be significantly less than ETRIMP_BURN_, despite both tasks having similar durations. In an on-duty setting, ETRIMP has been utilized to quantify the physiological load of different emergency call types [20]. In contrast, ETRIMP_TREAD_ and ETRIMP_BURN_ demonstrated lower physiological loads than average on-duty fire emergencies that included fire suppression and/or auto-extrication (74.33 ± 59.84) [20]. It is possible that the ETRIMP of a live-fire response is greater than the present tasks due to a longer average duration (42.75 ± 23.67 min) [20]. Interestingly, the magnitudes of ETRIMP_TREAD_ and ETRIMP_BURN_ are comparable to those elicited through high-intensity functional training among physically active populations [44]. Specifically, such training programs elicited ETRIMPs ranging from 19.8 to 77.7 AUs across durations of 4.06–17 min [44], which suggests that it is possible that a high-intensity training session approximately equal to the duration of the fire task in the present study (~11 min) may elicit a comparable ETRIMP (~49 AU). Due to the similar durations of TREAD and BURN, the difference between ETRIMP_TREAD_ and ETRIMP_BURN_ is likely the result of differences in HR responses across the intensity zones that comprise the task workloads and inform the underpinnings of the physiological differences in load experienced across tasks.

The distributions in time across the individual intensity zones were significantly different across the maximal lab- and field-based tasks in the present study. TREAD elicited even distributions in time for ZONE1–ZONE4, affirming that the graded nature of the test achieved consistent increases in HR responses across intensity zones. However, near the conclusion of the graded test, where participants approached their physiological maximum, participants elicited HR responses in ZONE5 for a length of time significantly greater than the lower zones, suggesting that participants were able to extend the amount of time working at the maximal zone until volitional exhaustion. It is possible that this is a result of perceived effort by the participants, where at the point of exhaustion in high-intensity exercise, the exercise tolerance for highly motivated subjects is limited by their perception of effort [45]. Unlike TREAD, the HR responses for BURN were unequally distributed across zones, where greater time was elicited for ZONE4 and ZONE5 versus ZONE1–ZONE3, reflecting substantially greater time spent at HR intensities greater than 80%HR_MAX_ rather than moderate intensities (i.e., <80% HR_MAX_). This is similar to the prior literature, where a simulated fire suppression elicited a greater HR_MAX_ response compared to an annual aerobic test in turnout gear for 84% of participants, thus demonstrating that fire suppression elicits cardiocirculatory strain not sufficiently reflected in a medical or lab examination [36]. Altogether, it is likely that the controlled, graded nature of the treadmill test influenced the uniformity in time spent across ZONE1–ZONE4, which was uncontrolled in the field-based task and likely contributed to the exacerbated time spent above 80%HR_MAX,_ in addition to other task-specific factors.

There are several task-specific factors that likely influenced the HR responses in the field-based fire suppression task to a greater extent than the laboratory-based treadmill test, thereby eliciting a greater magnitude of workload. It is likely that the inherent nature of the fire suppression tasks (i.e., stair climbing, hose advancement), in addition to the instructions to perform them at an emergency pace to simulate a realistic environment, significantly influenced the HR responses of the field-based task, like those similarly demonstrated in simulation scenarios [46,47,48]. Additionally, the inclusion of exercising in encapsulating PPE, and breathing from a SCBA, adds an additional weight of approximately 22.4 kg [9]. Donning PPE and SCBA in temperate conditions has been shown to increase FF HR responses during a graded treadmill protocol [49], yet not elicit significantly different body temperature responses [50], suggesting that the weight of the PPE and SCBA may partially contribute to the heightened HR responses during BURN over TREAD in the present study. However, it is also possible that the elevated HR responses during the fire suppression with PPE were compounded by the heated environment (average at Shoulder Height: Start [173.22 °C], End [112.94 °C]). Bruce-Low et al. [51] demonstrated that PPE and SCBA in the presence of a live fire can increase body temperature responses and elicit a 39.8% increased HR response, thus suggesting that a lack of heat dissipation from PPE can elevate cardiovascular response. It is likely that the combined load (i.e., weight and encapsulation) of the PPE and SCBA, with the high-temperature conditions of the fire suppression, supported an elevated HR response and resulted in a workload of a larger magnitude for the field-task. 

Due to ETRIMP being historically utilized to quantify the load of training stimuli, which subsequently influence performance and fitness measures [21,52], links between measures of fitness and how they inform task workload responses remains relatively unexplored. Interestingly, the aerobic fitness and body composition of the FFs in the present study demonstrated relationships to the elicited workloads of both TREAD and BURN in unique ways. ETRIMP_TREAD_ was negatively related to BF% and positively related to VO_2PEAK_, suggesting that individuals with better body composition profiles and/or greater peak rates of oxygen consumption were able to work to a greater maximal workload. Though BF% and VO_2PEAK_ were related to ETRIMP_TREAD_ as an overall measure of load, there were no significant relationships between either measure and the duration of time within ZONE1–ZONE5, which is likely due to the lab-controlled, graded nature of the test, where each individual progresses through pre-determined stage durations at a rate uninfluenced by one’s physiological (i.e., heart rate) response to the load. As such, all participants begin TREAD at the same grade and speed; however, this is a lower relative intensity for more-fit individuals, thus allowing them to continue exercising for a longer duration before reaching a maximal state. Accordingly, this likely accounts for the similar durations in ZONE1–ZONE5, despite a greater ETRIMP_TREAD_ for individuals with a lower BF% or greater VO_2PEAK_. These findings align with research demonstrating a strong negative relationship between BF% and total test duration (*r* = −0.704), as well as a strong positive relationship between VO_2PEAK_ and total test duration (*r* = 0.863), for a maximal treadmill test that followed the same treadmill protocol as this study [53].

On the contrary, ETRIMP_BURN_ was unrelated to BF% and VO_2PEAK_, suggesting that in a controlled fire, participants with higher levels of obesity and/or lower peak rates of oxygen consumption worked at an overall load similar to those with enhanced body composition or aerobic capacity profiles. Interestingly however, the distribution of time spent across the intensity zones was related to fitness, where FFs with a lower BF% and/or greater aerobic capacity accumulated less overall time in ZONE5 and instead spent more time in ZONE3–ZONE4 and ZONE3, respectively. These findings are in alignment with Windisch et al.’s [54] demonstration of a strong, negative relationship (*r* = −0.693) between peak aerobic capacity and average HR during a simulated firefighting exercise with heat and flashover exposure. Thus, regardless of fitness, FFs will perform the job tasks required in a fire suppression setting; however, less time is spent near a maximal level of exertion for those with higher levels of fitness. Prior research demonstrates that FFs with lower aerobic fitness capacities are more likely to experience cardiovascular abnormalities during maximal exercise [55], and obesity status is linked to the heightened occurrence of on-duty cardiac fatalities [13]. Furthermore, Poplin et al. [12] reported that lower aerobic capacities have been related to greater injury rates generally, including musculoskeletal strains and sprains, and Ras et al. [56] demonstrated that FFs with an increased BF% have a greater risk of musculoskeletal injury. Accordingly, suggestions have been made that the risk of cardiac events in less-fit individuals is heightened because more-fit FFs experience less strain when completing the same work [2]. This notion is supported by the fitness relationships (i.e., VO_2PEAK_ and BF%) to fire suppression intensity in the present study. Thus, the quantification of the cardiovascular cost to complete FF-specific tasks should be strongly considered when strategizing to maximize FF health through minimizing cardiovascular and/or musculoskeletal injury.

Finally, it should be noted the BMI did not demonstrate any significant relationships between the ETRIMP or ZONE1–ZONE5 responses for TREAD or BURN, thus suggesting that the distribution of mass may not inform FF workload. These results align with Barry et al.’s [37] demonstration that BMI is unrelated and unpredictive of VO_2PEAK_ in FFs. Similarly, prior research indicates the BMI is unrelated to FF autonomic nervous system recovery [38] and as such, supports the overall notion that the BMI may not be as sensitive as BF% when examining body composition influence on FF performance as it relates to cardiovascular measures.

The results of this study must be interpreted within the limitations of the study. Due to the BURN exercise being performed in groups, it is possible that the workload was not standardized or evenly distributed between FFs. As such, there is potential that a discrepancy may have existed in the physical burden placed between the FFs within this sample, which may account for the relatively large range (i.e., standard deviations) of the zones observed during the BURN, but not during the TREAD. However, this group-work design towards completing the BURN task is ecologically valid, as it reflects the team-based strategies utilized during live-fire suppression. Additionally, the single bout of exertion required to complete the BURN task may not reflect the actual demands of a live suppression that often require multiple work cycles from a single crew, which have been demonstrated to lead to further heart rate increases with each successive bout [57,58]. Similarly, the psychological stress experienced during a training fire suppression may not reflect the stressful demands of a live suppression that can include high-stakes scenarios (e.g., victim entrapment), and future researchers should examine the influence of these potential differences on physiological workload responses. An additional limitation to consider is the small representation of female FFs within this sample (n = 2). However, approximately only 9% of career FFs are female [59] and as such, this sample (e.g., 13% female) is reflective of the current state of the fire service profile. Lastly, future researchers should examine how other fitness characteristics (i.e., muscular performance) influence maximal task workload responses and if differences exist between laboratory- and field-based tasks.

## 5. Conclusions

The primary findings of this study uniquely demonstrate that within an FF population, the physiological workload of a maximal treadmill test is significantly less than a field-based fire suppression exercise, suggesting that while a maximal treadmill test reflects FF fitness, this traditionally utilized laboratory-based test does not adequately reflect the workload required to perform fire-suppressive work. Accordingly, practitioners should consider utilizing laboratory- and field-based measures in tandem, to holistically understand FF health, fitness, and job readiness. Further, due to the maximal lab-based and field-based tasks in the present study being utilized to inform FF readiness for duty, yet exhibiting seemingly lower physiological workloads than those of a live on-duty fire suppression [20], future researchers should examine the efficacy of utilizing either maximal task to indicate a readiness to meet the workload demands of live-fire emergency calls. Finally, regardless of aerobic fitness and/or body composition, the present study demonstrated that FFs perform the physiological workload necessary to complete the tasks of a fire suppression exercise. However, less-fit FFs spend significantly greater amounts of time at higher cardiovascular intensities than more-fit individuals during fire suppression. Specifically, during fire suppression, individuals with higher percentages of fat mass and/or and lower aerobic capacities accumulated greater amounts of time at intensities above 90%HR_MAX_. These findings suggest that the relative intensity of the cardiovascular system to complete the objective work of fire suppression is influenced by fitness and should be taken into consideration when implementing individual preparation and recovery strategies to best mitigate job-affiliated injury and health risks.

## Figures and Tables

**Table 1:** Average percent of total time spent in workload intensity zones for maximal tasks.

	TREAD	BURN
ETRIMP (AU)	35.87 ± 6.17	49.13 ± 9.64
ZONE1 (%)	17.19 ± 11.0	00.00 ± 00.00
ZONE2 (%)	15.75 ± 8.53	2.02 ± 6.21
ZONE3 (%)	17.87 ± 3.08	9.98 ± 17.43
ZONE4 (%)	20.91 ± 5.90	26.09 ± 21.26 + ^§^
ZONE5 (%)	28.28 ± 8.63 ^§,ф^	61.90 ± 33.44 + ^§,ф^

Mean ± SD; +, significantly different (*p* < 0.05) from ZONE1 within task; §, significantly different (*p* < 0.05) from ZONE2 within task; ф, significantly different (*p* < 0.05) from ZONE3 within task.

**Table 2:** Relationships between workload and fitness characteristics.

		BMI	BF%	VO_2PEAK_
TREAD	ETRIMP (AU)	−0.380	−0.624 *	0.741 **
	ZONE1 (%)	0.105	0.127	−0.265
	ZONE2 (%)	0.279	0.126	−0.079
	ZONE3 (%)	−0.272	−0.411	0.450
	ZONE4 (%)	0.049	−0.066	0.212
	ZONE5 (%)	−0.346	−0.094	0.110
BURN	ETRIMP (AU)	0.092	0.246	−0.199
	ZONE1 (%)	---	---	---
	ZONE2 (%)	0.045	−0.196	0.451
	ZONE3 (%)	−0.237	−0.580 *	0.597 *
	ZONE4 (%)	−0.118	−0.657 **	0.284
	ZONE5 (%)	0.190	0.756 **	−0.576 *

*, *p* < 0.05; **, *p* < 0.001; ---, not possible to calculate.

## Data Availability

The data that support the findings of this study are available on request from the corresponding author. The data are not publicly available due to information that could compromise research participant confidentiality.
